# A Mindfulness-Based Lifestyle Intervention Among Economically Marginalized Caregiver-Preschooler Dyads: Feasibility, Acceptability, and Satisfaction

**DOI:** 10.1007/s12310-025-09767-w

**Published:** 2025-05-26

**Authors:** Jiying Ling, Autumn Ashley, Nagwan Zahry, Tsui-Sui A. Kao, Charis L. Wahman, Kenneth Resnicow, Lorraine B. Robbins, Jean M. Kerver, Nanhua Zhang

**Affiliations:** 1 College of Nursing, Michigan State University, East Lansing, USA; 2 Department of Communication, University of Tennessee, Chattanooga, USA; 3 Department of Counseling, Educational Psychology and Special Education, Michigan State University, East Lansing, USA; 4 Division of Epidemiology & Community Health, University of Minnesota, Minneapolis, USA; 5 Department of Epidemiology and Biostatistics, Michigan State University, East Lansing, USA; 6 Division of Biostatistics and Epidemiology, Department of Pediatrics, Cincinnati Children’s Hospital Medical Center, University of Cincinnati College of Medicine, Cincinnati, USA

**Keywords:** Mindfulness, Mental health, Childcare, Child, Family, Feasibility

## Abstract

Mindfulness-based interventions are increasingly recognized for their positive impact on children’s physical, mental, emotional, and behavioral health. However, no mindfulness-based lifestyle interventions have focused on improving both the physical and mental well-being of economically marginalized preschoolers. Therefore, this one-group study aimed to examine feasibility, acceptability, and satisfaction of a 5-week mindfulness-based lifestyle intervention among preschoolers, caregivers, and childcare teachers. Caregiver-preschooler dyads were recruited from one urban and one rural Head Start childcare center. Outcome data were collected at baseline only, while evaluation data were obtained following the intervention. The intervention included three components: a school-based mindful eating and movement learning for preschoolers; a home-based caregiver training on mindful eating, movement, and parenting; and a school learning and home practice connection in mindfulness. Nineteen preschoolers, 18 caregivers, and three teachers participated. The enrollment rate was 40.4%. Attrition was 0% among preschoolers and 5.6% (*n* = 1) among caregivers (one caregiver passed away). Baseline data collection completion rate was 100%, with 88.9% (*n* = 16) caregivers and 84.2% (*n* = 16) preschoolers having valid ActiGraph data. Hair sample parental consent rate was 57.9% (*n* = 11), while sample collection rate was 90.9% (*n* = 10). Intervention implementation fidelity was excellent. Average intervention participation rates were 83.2% (*n* = 16), 72.2% (*n* = 13), and 55.6% (*n* = 10) for the preschooler component, caregiver meeting, and social media-based caregiver component, respectively. Acceptability and satisfaction with the intervention were high among caregivers and teachers. The results support the feasibility, acceptability of, and satisfaction with the 5-week intervention among preschoolers and caregivers from economically marginalized families and childcare teachers.

**Supplementary Information:**

The online version contains supplementary material available at 10.1007/s12310-025-09767-w.

## Introduction

Mental, emotional, and behavioral (MEB) disorders are highly prevalent among young children in the USA, affecting one in every six children aged 2–6 years (Charach et al., 2020). Internationally, about 8% of children and 15% of adolescents have a mental disorder, with suicide being the third leading cause of death among 15–29-year-olds (World Health Organization, 2025). Early childhood MEB disorders can manifest as disruptive problems, such as aggression, attention deficit hyperactivity disorder (ADHD), anxiety, and depression (Gilliom & Shaw, 2004). These disorders can persist into adolescence and even adulthood (Bitsko et al., 2022; Ogundele, 2018), resulting in adverse physical, mental, cognitive, and social outcomes, including obesity, cardiovascular diseases, elevated suicide risk, panic disorder, and substance abuse disorders (Bitsko et al., 2022; Miron et al., 2019; National Research Council and Institute of Medicine Committee on the Prevention of Mental Disorders and Substance Abuse Among Children, 2009). Thus, mitigating the underlying causes of MEB disorders in childhood and intervening to prevent the escalation of dysfunctional behaviors and long-term consequences is a public health priority (American Academy of Pediatrics, 2021).

Beyond physiological, neural, and genetic factors, environmental influences at home and in childcare centers play a significant role in escalating the behaviors and outcomes associated with MEB disorders among preschoolers (Egger & Angold, 2006). Specifically, family-based risk factors, including caregivers’ (i.e., parents or legal guardians) poor mental health and parenting practices can adversely impact children’s MEB well-being (Cree, 2018; Ling et al., 2020). For example, caregivers with high levels of stress and distress are more likely to have preschoolers who exhibit similar problems (Ling et al., 2020). Further, caregivers struggling with poor mental health engage in less effective parenting practices, including insufficient regulation of their preschoolers’ physical activity participation and healthy eating behaviors (Jang et al., 2019). This occurrence is particularly true for children from low-income and economically marginalized families (LIEM) due to caregivers often struggling with mental health issues resulting from poverty-related stressors, such as financial challenges and food insecurity (Cree, 2018; Kimbro & Denney, 2015; Yoshikawa et al., 2012).

With regard to childcare centers, preschoolers receiving quality childcare (e.g., daily learning activities and play-based programs) are more likely to develop positive MEB and physical health (Charrois et al., 2020; Gomajee et al., 2018). A crucial component of childcare quality is well-trained teachers who can create a nurturing learning environment that enhances children’s cognitive, emotional, and behavioral skills (Blewitt et al., 2020). Conversely, low quality childcare can amplify early risk factors for MEB disorders, especially among children from LIEM backgrounds (Gomajee et al., 2018; Wilhelmsen et al., 2023).

Health disparities in MEB disorders have been documented, underscoring the interplay among low socioeconomic conditions, lifestyle factors, and physical and mental well-being (Kimbro & Denney, 2015; Yoshikawa et al., 2012). One example is food insecurity, which is associated with MEB outcomes, such as depression and anxiety among children from LIEM backgrounds (Shankar et al., 2017). Furthermore, poverty is associated with unhealthy lifestyle factors, including poor dietary patterns and lack of physical activity; this can increase children’s vulnerability to MEB disorders, as well as health issues (e.g., obesity; Kimbro & Denney, 2015; Yoshikawa et al., 2012). Not only are children from LIEM backgrounds at heightened risk for developing MEB disorders, but they also encounter barriers to accessing MEB professional health services (Cree, 2018; Whitney & Peterson, 2019). Thus, addressing disparities in MEB disorders requires multi-level interventions that target both the home and childcare environments for preschoolers and caregivers from LIEM backgrounds.

### Mindfulness-Based Interventions in Early Childhood

Mindfulness-based interventions (MBIs) are increasingly recognized for their positive impact on children’s MEB and physical health (Bockmann & Yu, 2023). *Mindfulness* can be defined as the conscious act of being fully present and attentive to the current moment through embracing one’s thoughts, emotions, and behaviors with curiosity and acceptance (Bishop et al., 2004). MBIs can benefit children as young as 3 years old, leading to improved self-regulation and social-emotional functioning (Bockmann & Yu, 2023; Sun et al., 2021); it can also assist in enhancing early childhood educators’ implementation practices (Hatton-Bowers et al., 2022). However, the majority of MBIs have primarily targeted school age children and adolescents aged 6–18 years (Kander et al., 2024; Pickerell et al., 2023), with little attention to preschoolers, particularly those from LIEM backgrounds (Li-Grining et al., 2021; Ling et al., 2024a, 2024b).

Among a few MBIs designed specifically for preschoolers, all have yielded some promising results regarding emotional and behavioral outcomes. For example, a systematic review with 16 studies found that yoga and mindfulness interventions positively promoted preschoolers’ social-emotional learning, such as emotion regulation, attentional capacities, and prosocial behavior (Sun et al., 2021). However, all 16 studies were assessed as having moderate-to-high risk of bias, with sample sizes ranging from 23 to 325. Similarly, another review of 18 studies, with sample sizes ranging from 23 to 584, indicated that MBIs, including yoga-based MBI, mind–body awareness, and MBIs incorporating social-emotional learning, had mixed effects on improving emotion regulation, but demonstrated positive effects on enhancing behavioral regulation, including decreased hyperactivity and aggression (Bockmann & Yu, 2023). Although MBIs have shown positive effects on reducing stress and anxiety in children and adolescents (Dunning et al., 2022), their effects as universal interventions for improving preschoolers’ mental well-being are unknown.

Regarding lifestyle behavioral changes and obesity preventions, MBIs tend to focus more on improving eating behaviors than physical activity, especially among young children (de Lara Perez & Delgado-Rios, 2022). Overall, MBIs have demonstrated some positive effects in improving preschoolers’ dietary quality (Ling et al., 2024a, 2024b), mindful eating (Hong et al., 2018), and food liking (Schmitt et al., 2020). However, only about 18% of MBIs showed an effect in preventing childhood obesity (de Lara Perez & Delgado-Rios, 2022). Additionally, MBIs have been applied to promote physical activity participation and adherence through improving participants’ acceptance of activity-related discomfort thoughts and sensations (Salmon et al., 2010). For example, a recent systematic review with 14 randomized controlled trials (13 in adults and one in adolescents) found some positive effects of MBIs on physical activity outcomes (Schneider et al., 2019). Only one MBI was identified that focused on improving both healthy eating behavior and physical activity in 53 adolescents, and the intervention had significant effects on increasing physical activity, but not eating habits (Salmoirago-Blotcher et al., 2018). One plausible explanation for these mixed effects is the lack of parental involvement, particularly in promoting mindful eating practices at home.

To the best of our knowledge, no mindfulness-based lifestyle intervention has been developed or evaluated that focuses on improving both the physical and mental well-being of LIEM preschoolers and their caregivers. The current pilot feasibility study contributes to the emerging field of MBIs tailored to preschoolers and their caregivers by assessing the feasibility of a 5-week Food-Body-Mind mindfulness-based lifestyle intervention designed for caregiver-preschooler dyads from LIEM urban and rural backgrounds. Guided by widely recognized assessment standards (Feagans Gould et al., 2016; Kechter et al., 2019), this feasibility study examines enrollment and attrition, data collection participation, intervention delivery fidelity, intervention participation, acceptability, and satisfaction with the intervention among preschoolers, caregivers, and childcare teachers. Results from this feasibility study will inform the conduct of a cluster randomized controlled trial to evaluate the effects of the full 16-week intervention on improving MEB and physical health among LIEM preschoolers and their caregivers.

## Methods

### Study Design and Sampling

A one-group, pre–post-feasibility study was conducted among one urban and one rural Head Start childcare center from the Midwestern USA, where teachers expressed interest in participating. The rationale for selecting one urban and one rural center is to obtain a representative sample across diverse environments because the full-scale clinical trial aims to test an intervention in both urban and rural settings. All enrolled participants received a 5-week mindfulness-based lifestyle intervention. A timeline of five weeks was based on resource and time constraints during the initial 1-year phase, with a primary focus on process evaluation and implementation feasibility. Outcome data were collected at baseline only, and evaluation data were obtained immediately after the intervention. Both the feasibility study and the full-scale clinical trial evaluating the 16-week intervention were registered on clinicaltrials.gov (NCT05964218, NCT06597474, respectively). The entire study was approved by the Michigan State University Biomedical and Health Instructional Review Board (approval ID: STUDY00009256), and Head Start organizations endorsed the study by signing a memorandum of understanding.

Caregiver-preschooler dyads were recruited using a non-random convenience sampling approach through multiple strategies: (1) classroom teachers distributed both electronic and hard-copy recruitment flyers to families; (2) teachers shared contact information of interested caregivers, who had given permission, with the research team to complete enrollment; and (3) the study project manager conducted in-person recruitment during morning child drop-offs and afternoon pick-ups. Each family received a $10 Amazon e-gift card after completing the study’s screening and enrollment survey, regardless of their decision to participate.

Preschoolers were eligible to participate when they were 3–5 years old and enrolled in a full- or part-day Head Start childcare program. Caregivers were eligible for enrollment if they were the primary adult caregiver for the preschooler, had at least weekly access to the Internet, and were willing to use Facebook or the study’s private website to participate in intervention activities for 5 weeks. Preschoolers were excluded when they had a motor disability or impairment (e.g., cerebral palsy, spinal cord injury) preventing them from participating in any physical activity, a diagnosed medical condition (e.g., phenylketonuria) precluding them from any dietary change, or a diagnosed developmental disorder (e.g., autism Level 3) causing severe difficulty with communication. Caregiver informed consent and child verbal assent (when the preschooler was 5 years old) were obtained before any data collection. Preschoolers’ verbal assent was initially obtained and recorded in the online enrollment survey with assistance from their caregivers and later confirmed by the research team during in-person data collection. All childcare teachers were invited to participate in data collection after providing informed consent.

### Intervention

Anchored in the Actor–Partner Interdependence Model (Cook & Kenny, 2005), the Allostatic Load Model (McEwen, 2000), and the Transactional Theory of Stress and Coping (Lazarus & Folkman, 1987), the 5-week Food-Body-Mind intervention included three components: (a) school-based mindful eating and movement learning for preschoolers; (b) home-based caregiver training on mindful eating, movement, and parenting; and (c) school learning and home practice connection in mindfulness. The Actor–Partner Interdependence Model serves as the fundamental framework for the intervention by reinforcing the bidirectional influences between preschoolers and caregivers. The Allostatic Load Model and the Transactional Theory of Stress and Coping connect stress and coping to health outcomes. Drawing from these theories, the intervention targeted the connections among physical activity, dietary intake, and mental health to improve both preschoolers’ and caregivers’ adaptive coping, such as mindful eating, movement, and parenting to enhance their overall health. In childcare classrooms, preschoolers learned mindful eating via weekly fruit/vegetable (F/V) taste test activities and mindful movement of yoga poses and deep breathing exercises following the “Eat & Walk My ABCs” curricula taught by childcare teachers who completed a curriculum teaching training. Two lessons were taught by teachers each week, with each lesson lasting for about 20 min. After each lesson, preschoolers created letters using stickers to share what they had learned in school and what they wanted to continue at home with their caregivers. The letters were then delivered to caregivers via text messaging to encourage them to continue practicing mindful eating and movement at home with their preschoolers.

During Week 1, one group caregiver meeting was hosted via Zoom. The session provided a 20-min guided yoga practice, an overview of the intervention, and ways of incorporating mindful eating practice at home. The meeting was held during two time slots: Wednesday at noon and Saturday at 10:00 a.m. During Weeks 2–5, caregivers joined the study’s private website and/or a private Facebook group to learn skills on mindful eating, movement, and parenting. Caregivers had the option to engage in intervention discussions either through the study’s website or the private Facebook group because some caregivers might have privacy concerns about using Facebook (Titgemeyer & Schaaf, 2022). A flyer and video containing the relevant information was posted by the intervention coordinator each week to the Facebook private group, as well as the study website. Caregivers were reminded to complete four tasks via the study’s website and/or the private Facebook group each week: (1) create a healthy family (post about a healthy meal made for the family or a physical activity they helped the child engage in, or ways of practicing mindful eating and movement at home); (2) raise a happy family (post about a life management tip they used to manage life and stress or a strategy used to improve the parent–child relationship); (3) leave a positive comment (positively respond to one other person’s post to build a virtual supportive community); and (4) take a quiz through the study’s website (take a brief online quiz to reinforce information and strategies learned). In addition, caregivers received three standardized motivational text messages each week (one on Monday, Wednesday, and Friday, respectively) about parenting, family, lifestyle change, habit formation, stress management, and mindful practice. The motivational text messages were automatically delivered via the Twilio communication platform and did not request caregivers to provide a response.

#### Intervention Implementation Fidelity Evaluation

Independent process evaluators conducted observations to evaluate the fidelity of the (1) child curricula teaching by childcare teachers; and (2) caregiver meeting delivery by the intervention coordinator. For each classroom, two process evaluators observed two lessons (one on Eat My ABCs, the other on Walk My ABCs) and completed an investigator-developed “Preschooler Program Observation Form” to evaluate the adherence to the curricula. The observation form was tested in a prior study (Ling et al., 2024a, 2024b) and contained 22 close-ended questions. The sum adherence score (0–6) of the first six questions assessing lesson duration, flow, pace, engagement, content, and mindfulness principles was based on response choices of yes/no, fluent/disfluent, appropriate/too slow/too fast, rarely/sometimes/often. The average evaluation score (1–4) of the remaining questions evaluating preschoolers’ participation, engagement, teaching style, and lesson content was based on response choices of strongly disagree, disagree, agree, strongly agree. Similarly, two process evaluators observed two offered caregiver meetings for Meeting 1 and completed a 10-item investigator-developed “Caregiver Meeting Observation Form,” which assessed caregivers’ participation, engagement, yoga teaching, presentation style, and mindful eating content. Each item had four response choices: strongly disagree (1), disagree (2), agree (3), and strongly agree (4). Average evaluation scores (1–4) from process evaluators were calculated to assess intervention implementation fidelity. The intervention coordinator shared the consolidated evaluations and feedback from the process evaluators with childcare teachers.

### Data Collection and Measures

#### Baseline Outcome Data Collection

Baseline outcome data collection included two components: (a) an online survey and (b) in-person data collection. After enrollment and screening, eligible caregivers received a link to the baseline survey via text messaging and/or emails aligning with participants’ preferred contact methods. The survey measured preschoolers’ behavioral health and emotional well-being (sadness, fear, anger, positive affect); caregivers’ stress, anxiety, depression, mindfulness, and coping; caregiver-preschooler dyads’ emotional eating and relationship; and families’ home environment and food insecurity. To assess participants’ attention when completing the long online survey of 289 questions, an attention check question was included in the middle of the survey. To achieve better response rates, the study data collection team applied a structured reminder system using each caregiver’s preferred contact methods, such as text messages, phone calls, or emails: (a) first reminder to caregivers after 3 business days; (b) second reminder to both caregivers and classroom teachers (classroom teachers were asked to help remind caregivers about data collection) after 1 week; and (c) third reminder to classroom teachers after 2 weeks. Furthermore, we implemented additional strategies to enhance the online survey completion rate. These strategies included providing an introduction at the beginning of each instrument, utilizing simple close-ended questions, employing large fonts, allowing later completion, integrating a constant speed progress bar and a back button throughout, generating reminders for unanswered questions, and documenting incomplete survey responses, aligning with suggested strategies in the literature (Liu & Wronski, 2017).

After completing the online survey, caregivers were scheduled for in-person data collection at the childcare centers. During in-person data collection, the study data manager and trained data collectors measured dyads’ height, weight, percent body fat, skin carotenoids, and caregivers’ blood pressure. Each dyad then received two ActiGraph accelerometers to wear for a week. For a subgroup of preschoolers with parental consent for hair sample collection (this data collection was optional), a small amount (about half of the diameter of a pencil) of 3-cm hair was cut at their posterior vertex of head. The hair samples were mailed to the Child Study Center Lab at Yale University to extract cortisol concentration using the enzyme immunoassay approach (Meyer et al., 2014). For in-person data collection, we offered make-up and home-visiting options to address barriers, such as transportation constraints and scheduling conflicts. Additionally, we provided itemized monetary incentives (cash or e-gift cards: $40 for online survey; $40 for in-person data collection; $20 for having valid ActiGraph data; and $30 for providing a child hair sample) for each data collection activity to cover transportation and childcare costs, as well as to compensate participants for their time. Table 1 demonstrates the measures for each baseline outcome. All selected measures had established acceptable reliability and validity, with good reliability (0.57–0.96) in this study.

**Table 1:** Baseline outcome measurements

Variable	Measure and description	Response choices	Scoring	# Items	Cronbach’s a in the study
Preschooler					
Behavioral health	Preschool and kindergarten behavior scales-second edition (PKBS-2) (Merrell, 1994) with 2 subscales of social skills & problem behaviors	Never, rarely, sometimes, often	Sum	76	0.88
Chronic stress	Hair cortisol concentration (Meyer et al., 2014)	N/A		N/A	N/A
Sadness	Sadness parent report fixed form (Salsman et al., 2013)	Never or not true, sometimes or somewhat true, often or very true	*t*-score	7	0.63
Fear	Fear-over anxious parent report fixed form (Salsman et al., 2013)	Never or not true, sometimes or somewhat true, often or very true	*t*-score	6	0.66
Anger	Anger parent report fixed form (Salsman et al., 2013)	Never or not true, sometimes or somewhat true, often or very true	*t*-score	9	0.80
Positive affect	Positive affect parent report fixed form (Salsman et al., 2013)	Not at all, a little bit, somewhat, quite a bit, very much	*t*-score	9	0.95
BMI *z*-score	Calculated from height and weight using CDC growth charts (Centers for Disease Control & Prevention, 2021) *Height* Infant/Child/Adult ShorrBoard^®^ Stadiometer *Weight* InBody 270 body composition analyzer	N/A	*z*-score	N/A	N/A
% Body fat	InBody 270 body composition analyzer (Larsen et al., 2021)	N/A	%	N/A	N/A
Caregiver					
BMI	Calculated using (weight kg/height m^2^) (Centers for Disease Control & Prevention, 2022) Measures are the same as those used in preschoolers	N/A	Rate	N/A	N/A
% Body fat	InBody 270 body composition analyzer	N/A	%	N/A	N/A
Blood pressure	SunTech CT40 blood pressure device (Polo Friz et al., 2017)	N/A	Mm Hg	N/A	N/A
Stress	Perceived stress Scale (Cohen et al., 1983)	Never, almost never, sometimes, fairly often, very often	Sum	10	0.89
Anxiety	Neuro-QoL anxiety short form (National Institute of Neurological Disorders & Stroke, 2015)	Never, rarely, sometimes, often, always	*t*-score	8	0.95
Depression	Neuro-QoL depression short form (National Institute of Neurological Disorders & Stroke, 2015)	Never, rarely, sometimes, often, always	*t*-score	8	0.96
Anxiety/depression	Patient health questionnaire (Kroenke et al., 2009)	Not at all, several days, more than half of the days, nearly every day	Sum	4	0.81
Caregiver-preschooler dyad					
Physical activity (min/hour)	7-day ActiGraph wGT3X-BT accelerometer *Cut-points for preschoolers* sedentary (≤ 37 counts/15 s), light (38–419), moderate (420–841), & vigorous (≥ 842) (Pate et al., 2006) *Cut-points for caregivers* light (0–2689 counts/60 s), moderate (2690–6166), & vigorous (≥ 6167; Sasaki et al., 2011)	N/A	Min/hour	7-day	N/A
F/V intake	Skin carotenoid level via Veggie Meter (May et al., 2020) 2-question survey adapted from the National Institute of health eating at America’s table study all-day screener (Peterson et al., 2008)	1 time/month or less, 2–3 times/month, 1–2 times/week, 3–4 times/week, 5–6 times/week, 1 time/day, 2–3 times/day, 4–5 times/day, 6 or more times/day	Sum	2	0.90
Preschooler emotional eating	Child eating behavior questionnaire (CEBQ; Domoff et al., 2015) with 8 subscales of food responsiveness, enjoyment of food, emotional overeating, desire to drink, satiety responsiveness, slowness in eating, emotional undereating, and fussiness	Never, rarely, sometimes, often, always	Mean	35	0.77
Caregiver emotional eating	Three-factor eating questionnaire (TFEQ-R18V2; Karlsson et al., 2000) with 3 subscales of cognitive restraint, emotional eating, and uncontrolled eating	Definitely true, mostly true, mostly false, definitely false	Mean	18	0.83
Caregiver-preschooler relationship	Child-parent relationship scale (CPRS; Driscoll & Pianta, 2011) with 2 subscales of conflict and closeness	Definitely does not apply, not really, neutral not sure, applies somewhat, definitely applies	Mean	15	0.71
Caregiver mindfulness	Mindful attention awareness scale (Brown & Ryan, 2003)	Almost always, very frequently, somewhat frequently, somewhat infrequently, very infrequently, almost never	Mean	15	0.89
Caregiver coping	Brief COPE (Solberg et al., 2021) has 14 factors that can be grouped to 3 subscales of problem-focused, emotion-focused, and avoidant coping	Not at all, a little bit, a medium amount, a lot	Mean	28	0.88
Home environment	Family nutrition and physical activity screening Tool (Ihmels et al., 2009)	Never/almost never, sometimes, often, very often/always	Sum	20	0.82
Household food insecurity	U.S. household food security survey module (Frongillo et al., 1997)	Often true, sometimes true, never true	Sum	19	0.57
Demographics	Socio-demographic questionnaire (e.g., age, sex, race, family income, education, marital, employment)	N/A	N/A	12	N/A

#### Intervention Evaluation Data Collection

Immediately after the caregiver meeting, caregivers were asked to complete an investigator-developed online survey containing 10 close-ended and two open-ended questions on acceptability and satisfaction. One example question is “How satisfied are you with the yoga session? Very dissatisfied, dissatisfied, no opinion, satisfied, very satisfied.” After the 5-week intervention ended, all 17 caregivers and three childcare teachers were invited to complete an online evaluation survey and an individual interview. The teacher evaluation survey included questions on their demographics (e.g., age, sex, ethnicity, race) and evaluation regarding the preschooler curricula (14 close-ended and three open-ended questions). One example question is “The curriculum content is age appropriate for preschoolers. Strongly disagree, disagree, no opinion, agree, strongly agree.” The caregiver evaluation survey was comprised of 19 close-ended and four open-ended questions on the acceptability of and satisfaction with the program. An example question is “How acceptable is the program to you? Completely unacceptable, unacceptable, no opinion, acceptable, completely acceptable.” Following the study’s semi-structured interview guides (see supplemental document), adapted from the guide tested in our prior trial (Chen et al., 2025), trained interviewers conducted individual interviews with caregivers and teachers via phone or Zoom. Each interview was audio/video recorded and transcribed by a trained research assistant (RA). The transcripts were then independently verified by another RA.

### Data Analysis

Data cleaning, management, and analyses were performed using SPSS Statistics 28. All quantitative data were described with means, standard deviations, ranges, frequencies, and percentages (for categorical data). Cronbach’s alpha was calculated to assess the internal consistency of each outcome instrument at baseline. Qualitative data from caregivers and teachers were coded and analyzed using the conventional and directed content analysis (Elo & Kyngäs, 2008; Hsieh & Shannon, 2005) by two independent RA teams. Initially, the first author deductively developed a preliminary coding scheme based on the structure and domains of the interview guides. Following the scheme, two RA teams independently reviewed and coded the transcripts, met regularly to discuss coding decisions, and resolved discrepancies through consensus. After reaching consensus, they inductively analyzed the data to identify patterns and organize results within a categorization matrix that aligned with key areas of interest: perceptions of participant enrollment and intervention components (Edvard et al., 2020). This matrix allowed for systematic comparison across interviews and ensured that both anticipated and emerging themes were captured. Lastly, the first author synthesized the final qualitative evaluation results and integrated them with the quantitative data to enable a comprehensive mixed-methods interpretation of participant (caregiver and teacher) experiences. We used triangulation strategies, including multiple data collection methods (surveys and interviews), diverse data sources (caregivers and teachers), and multiple researchers involved in data collection and analysis, to enhance the validity and credibility of the results (Thurmond, 2001).

## Results

### Demographics

The demographic characteristics of the 19 participating preschoolers and 18 caregivers are demonstrated in Table 2. For the three participating childcare teachers, all were female, with a mean age of 43.7 years (SD = 6.5, range = 37–50). One teacher was Hispanic. Two were White, and one was multiracial. One teacher was single, and two were married or partnered. One had an annual family income of $30,000–$49,999, and two had annual incomes ≥ $50,000. All had at least a bachelor’s degree, and worked in Head Start for an average of 9.7 years (SD = 3.9, range = 7–14). Based on their self-reported height and weight, one was overweight (BMI = 25–29.9) and the other two had class I obesity (BMI = 30–34.9).

**Table 2:** Participants’ demographics

Variables	Study sample (*N* = 18 caregivers, 19 preschoolers)		Interview sample (*n* = 6 caregivers)	
Continuous	Mean	SD	Mean	SD
Preschooler age (months, range 38–59)	47.00	6.40	52.67	4.80
Caregiver age (years, range 20–65)	31.94	10.52	30.50	8.31
Number of children in family (range 1–5)	3.18	1.33	3.67	0.82

Categorical	*n*	%	*n*	%
Preschooler sex				
Male	9	47.4	3	50.0
Female	10	52.6	3	50.0
Preschooler ethnicity				
Hispanic	2	10.5	1	16.7
Non-Hispanic	17	89.5	5	83.3
Preschooler race				
White	12	63.2	3	50.0
Black or African American	3	15.8	1	16.7
Mixed	4	21.1	2	33.3
Caregiver sex				
Male	1	5.6	0	0
Female	17	94.4	6	100
Caregiver race				
White	14	77.8	5	83.3
Black or African American	3	16.7	1	16.7
Other (S. European)	1	5.6	0	0
Caregiver marital status				
Married/partnered	5	27.8	1	16.7
Separated/divorced/widowed	1	5.6	0	0
Single	12	66.7	5	83.3
Family annual income (USD)				
Under $20,000	11	61.1	3	50.0
$20,000–$29,999	3	16.7	2	33.3
$30,000–$49,999	1	5.6	0	0
$50,000 or above	3	16.7	1	16.7
Caregiver employment status				
Full time	7	38.9	3	50.0
Part time	2	11.1	0	0
Unemployed	9	50.0	3	50.0
Caregiver education level				
Less than high school graduate	4	22.2	2	33.3
High school graduate	8	44.4	3	50.0
Some college	3	16.7	0	0
Technical school/community college degree	1	5.6	1	16.7
Bachelor’s degree	2	11.1	0	0

### Feasibility

#### Enrollment and Attrition

**Fig. 1:**
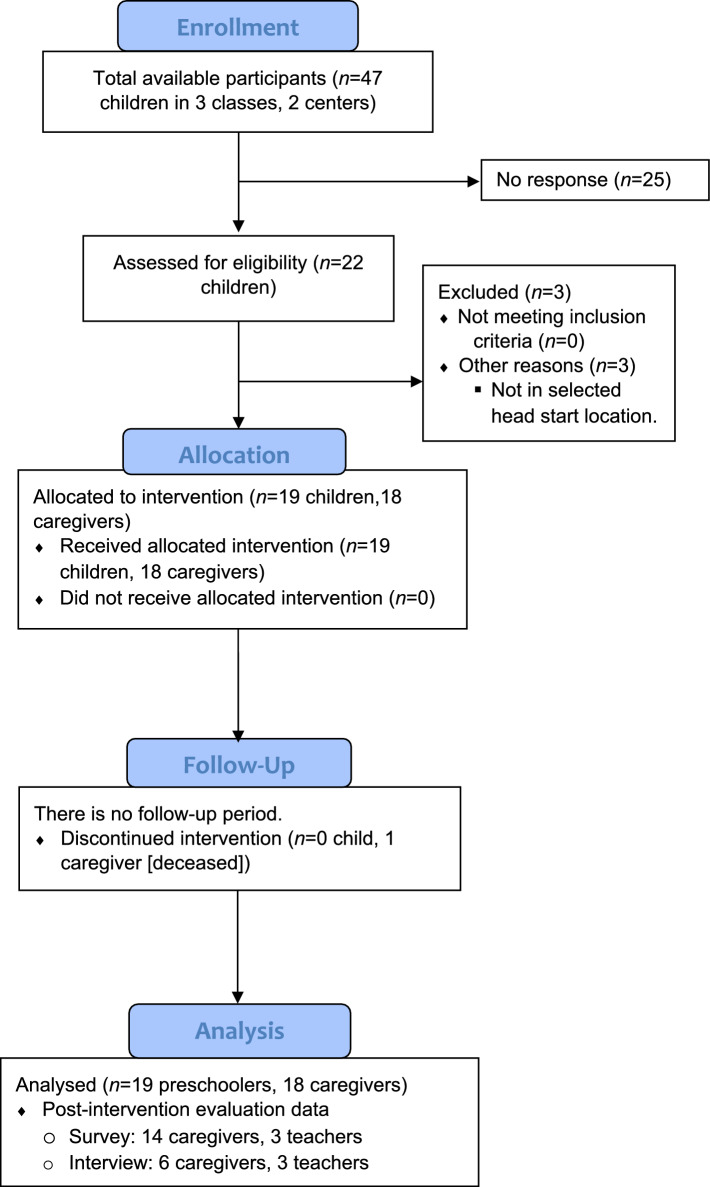
Study flow diagram

As demonstrated in Fig. 1, 22 (46.8%) of 47 preschoolers were assessed for eligibility. Nineteen preschoolers from 18 families were successfully enrolled in the study, resulting in an enrollment rate of 40.4%. According to interviews with six caregivers and three teachers, the recruitment flyer was “visually pleasing” and provided sufficient, yet easy-to-understand study information to help caregivers make an informed decision. Teachers also stated that the presence and continuous support of the research team facilitated the recruitment process, and they emphasized that incentives were imperative for enrolling this economically vulnerable population into research studies. One caregiver suggested delivering a recruitment presentation particularly at school parent meetings, while another recommended sharing testimonials from past participants. Attrition rate was 0% among preschoolers and 5.6% (*n* = 1) among caregivers; one caregiver was removed, as she passed away.

#### Data Collection

All 18 caregivers completed the baseline online survey with 100% passing the attention check. Caregiver in-person data (height, weight, percent body fat, blood pressure, skin carotenoids) completion rate was 100%, and preschooler in-person data (height, weight, percent body fat, skin carotenoids) completion rate was 94.7% (*n* = 18). Data were not collected for one preschooler due to the child choosing not to participate. All 18 caregivers and 19 preschoolers wore ActiGraph accelerometers for 7 days. All accelerometers were returned, and 16 (88.9%) caregivers and 16 (84.2%) preschoolers had valid data. Eleven (57.9%) preschoolers had parental consent for the optional hair sample collection, and 10 (90.9%) provided a hair sample. One sample was missing due to the child’s absence during in-person data collection. After the 5-week intervention, all three childcare teachers completed the intervention evaluation survey and an individual interview. Fourteen (82.4%) of 17 available caregivers completed the intervention evaluation survey with one caregiver missing some questions. No incentives were provided to caregivers for completing the evaluation survey. We interviewed six (33.3%) caregivers to reach data saturation; each caregiver was compensated with a $30 incentive.

#### Intervention Implementation Fidelity

For the school-based preschooler mindfulness curricula, we observed two lessons (one on “Eat My ABC” and the other on “Walk My ABC”) from each classroom. Based on the process evaluation, the sum adherence scores were 6 (SD = 0) and 5.67 (SD = 0.58) for the eating and walking lessons, respectively. The mean evaluation scores were 3.92 (SD = 0.10) for the eating lesson and 3.88 (SD = 0.08) for the walking lesson. We observed all caregiver meetings offered, and the mean evaluation score was 3.87 (SD = 0.19).

#### Intervention Participation

The average weekly attendance for the school-based preschooler component was 83.2% (*n* = 16) with a range of 68.4% (*n* = 13) to 94.7% (*n* = 18). The caregiver meeting attendance was 72.2% (*n* = 13). For the social media-based caregiver component, the average weekly participation (completed at least one task) was 55.6% (*n* = 10); ranging from 44.4% (*n* = 8) to 66.7% (*n* = 12) across 5 weeks.

### Acceptability and Satisfaction

#### Overall Evaluation

All 14 (77.8%) responding caregivers were satisfied with the entire intervention, found it acceptable, and stated it helped their families have a healthy and mindful lifestyle. Caregivers considered the intervention “a good program that gives the kids a little bit of an education while they have fun,” and agreed that “trying new foods” and “doing yoga poses” were their children’s favorite activities in the study. Similarly, all three teachers were satisfied with the intervention, and thought it met their expectations. Thirteen (92.9%) caregivers found the intervention materials easy to understand and helped to maintain a good relationship with their children. As a result of the intervention, one caregiver shared that they established a daily routine to practice yoga together every morning before school and another caregiver said that they were “more aware to keep their children more active than what they are already, and to throw more exercise in their normal activities.” Caregivers stated that there were “little to no barriers” that prevented them from participating in the entire feasibility study. All caregivers would continue to use what they had learned in the study and recommend to other parents. One caregiver shared that “being unpredictably sick with the flu” was a significant barrier to their participation in the intervention.

#### School-Based Preschooler Mindfulness Component

All three teachers agreed that the child curricula were informative, age appropriate, easy to understand, and had an appropriate length. All stated that the curricula increased preschoolers’ knowledge and skills of mindful eating, promoted consumptions of F/Vs, and increased preschoolers’ mindful movements. During interviews, teachers shared that they appreciated being able to offer fresh foods to children and that children really enjoyed exploring and tasting new foods.

Teachers also described the excitement the children felt in preparing for and doing yoga. One teacher discussed how children remembered the previous poses and requested to recap the old poses prior to learning new ones. The breathing balls were also well received by the three teachers. For example, one teacher shared: “The breathing ball was really helpful for the children, doing it and feeling it. Every part of their life, [a child] is controlled by somebody. Everyone is telling them what to do, so being able to do things like that, on their own, and have an object to help them, it is encouraging!” Through participating in the intervention, teachers stated that their children were regularly “talking about their breathing and their heart.” Teachers also noticed a “calming effect” on the classroom after participation; children continued to utilize the techniques they had learned, such as breathing and yoga poses, despite the end of the intervention. In addition, teachers felt supported by the research team and were quickly able to integrate the curricula into classroom routines. One teacher highlighted the challenge of “finding the space for 17 kids to lay down” during the intervention activities. During interviews, caregivers shared that the school-based curricula had helped their children stay engaged in learning, exposed them to different foods, and offered a point of connection between school and home through discussing daily new activities. One caregiver even stated, “I would love to see it expand into other schools because it is great!”.

#### Home-Based Caregiver Mindfulness Component

##### Social Media-Based Program

Five (83.3%) of six interviewed caregivers had very positive experiences with the Facebook component of the intervention. They enjoyed using the platform to “keep in touch with everything that was going on,” and completed weekly tasks with their children. All interviewed caregivers shared the perceived benefits of the program, including being positively influenced by seeing others’ posts and becoming more engaged with their children. For example, one caregiver shared that “I see other people working to be healthier, and if they’re doing it, there’s no reason I can’t.” Eleven (84.6%) of 13 survey caregivers shared that the weekly quizzes had helped them check their understanding of the content learned. Five (83.3%) of the six interviewed caregivers were satisfied with the weekly tasks and quizzes, felt they were achievable, and did not take too much time. By completing the tasks and quizzes, they felt more engaged with the program contents. One interviewed caregiver could not benefit much from the program because she was not comfortable with any social media platforms. Two (33.3%) caregivers suggested posting more activities they could do with their kids, organizing group meet-ups, and creating parent group text messaging for better engagement.

##### Caregiver Group Meetings

Eleven (91.7%) of 12 caregivers who completed the meeting evaluation survey were satisfied with the meeting content and found it helpful in improving knowledge and skills of practicing mindful eating at home. The caregiver who was dissatisfied with the meeting stated, “I enjoyed the meeting, I do not think I could have planned it any better,” but expressed frustration due to repeatedly losing connection. Nine (75%) were satisfied with the guided yoga practice, and about one quarter requested more yoga exercises. For example, one caregiver shared that “The yoga exercise provides a great interaction with my child because we don’t interact like that as a family very often.” Two caregivers had no opinion on the yoga session because they were unable to participate—one due to family members being sick back-to-back and the other due to time constraints from being a single parent and working. While make-up sessions were provided to both caregivers individually, the yoga session was not included. Five (83.3%) of the six interviewed caregivers (one did not attend the meeting due to illness) stated a beneficial effect of the meeting to promote mindful eating. They described the meeting as “helpful,” “relatable,” and “educational.” They appreciated the convenient virtual format and enjoyed learning about the different levels of hunger. The biggest barriers preventing caregivers from attending the group meeting were busy schedules due to work or being a single parent, unreliable Internet services, or illnesses among themselves and family members.

##### Weekly Motivational Text Messaging

Ten (76.9%) of the 13 caregivers (two caregivers had no opinion) who provided evaluation data stated that the weekly motivational text messages encouraged them to make healthy and mindful changes each day. Two caregivers felt that receiving three messages per week was overwhelming; one suggested sending messages earlier in the day rather than at noon. Consistently, all six interviewed caregivers enjoyed receiving motivational text messages that offered them “a healthy mindset” in their daily lives. All interviewed caregivers liked the idea of continuing the motivational text messaging once a week for 12 months.

#### *School Learning and Home Practice Connection *via* Child Letters*

All surveyed caregivers perceived the child letters as helpful for knowing about their children’s learning in school. Twelve (92.3%) caregivers discussed the letters with their children and even purchased and prepared foods according to the child letters. One caregiver selected “no opinion” because she felt the child program was not adequately explained to her during the program orientation. All caregivers shared that they had practiced the mindful activities (e.g., yoga, deep breathing) illustrated on child letters at home. During the interviews, caregivers shared that they enjoyed receiving the letters every week and learning about their children’s eating and mindful behaviors at school. Some caregivers stated that they were surprised to know their children tried and even “enjoyed” some new foods, because they had assumed their children were picky eaters. Caregivers shared how children would be excited to discuss the foods they tried, describing the sounds of a “snap” or “crunch” or the “little balls” shape as they discussed new foods. One caregiver shared her experience with her daughter: “It was so cute to see her every day when she got off the bus because she was so excited to tell me things, like: Mom, I tried this today and I liked it, or I tried that, and it was nasty.”

## Discussion

This study evaluated the feasibility, acceptability, and satisfaction of a 5-week mindfulness-based lifestyle intervention among preschoolers and caregivers from LIEM families, as well as childcare teachers. The successful enrollment (40.4%), low attrition (0%), high data collection completion (100%) and validation (84.2%–88.9%) rates, excellent intervention implementation fidelity, and great intervention participation (55.6–83.2%) support the feasibility of the study procedures and intervention. Evaluations from both childcare teachers and participating caregivers were very positive, supporting their acceptability of and satisfaction with the intervention. The sample demographics are very comparable to the state demographics, with 8.8% of children being Hispanic and 16.1% as Black (Michigan League for Public Policy, 2023).

The study’s enrollment rate of 40.4% was much higher than the participation rates of 17.1–28.6% achieved in previous clinical trials with LIEM families to promote healthy lifestyles (Ling et al., 2024a, 2024b; Ling et al., 2024a, 2024b). Possible reasons are the effective and appealing recruitment flyer, as well as the appropriate monetary incentives compensating participants for their time and efforts. These strategies were also found to be effective in recruiting participants in other clinical trials (Caldwell et al., 2010).

The high baseline data completion and validation rates of wearing ActiGraph are very encouraging in comparison with the 62.6% compliance rate reported in literature (Tudor-Locke et al., 2015), especially considering the significant time demands (on average 111 min for caregivers to complete the baseline survey) and in-person commitments (about 30 min for in-person data collection) from both preschoolers and their caregivers. These encouraging results may be due to the multiple strategies applied to mitigate challenges to data collection participation, such as time commitment and trust issues (Nathe et al., 2023). Thus, future community-based studies may consider some of the strategies to increase data collection completion and quality among both preschoolers and caregivers, such as adopting participants’ communication preferences, implementing a structured reminder system, accommodating participants’ specific needs by offering make-up and home-visiting options, and actively engaging community stakeholders (e.g., childcare teachers).

The 5-week pilot intervention demonstrated excellent delivery fidelity with an average evaluation score of 3.9, indicating strong adherence to the preschooler mindfulness curricula regarding lesson duration, pace, teaching style, content, and engagement. This accomplishment may contribute to our effective teacher training approach developed and tested in a previous trial with 23 teachers (Ling et al., 2024a, 2024b). Our teacher training contained a PowerPoint-guided interaction session, a lesson demonstration, and recorded lesson videos. Moreover, the strong support from the research team facilitated the implementation of the child curricula by childcare teachers.

The excellent intervention delivery fidelity may have resulted in the impressive intervention participation rates among preschoolers and their caregivers. The average weekly attendance of 83.2% for the preschooler component reflects the typical childcare attendance rates among preschoolers. As Head Start is a federally funded childcare program designed to support the holistic growth of young children from economically marginalized families, it has policy and procedures requiring childcare programs to meet a monthly attendance standard of 85% (U.S. Department of Health & Human Services, 2016). Our intervention attendance rate was slightly below the required monthly attendance standard, possibly due to prevalent illnesses (e.g., upper respiratory infections; hand, foot and mouth disease) and the challenging cold weather in February and March in the Midwestern USA. The high attendance rate of the school-based preschooler component highlights the benefits of implementing universal interventions in a school setting where children feel safe to learn and explore (Greenberg & Abenavoli, 2017).

The caregiver weekly participation rates of 44.4–72.2% fall within the range of 14–95% reported in studies engaging parents in behavioral parent training to treat disruptive behavior disorders in children (Chacko et al., 2016). The intervention’s flexible delivery methods (virtual meeting, social media platform [Facebook], private website) may be key factors contributing to the high caregiver participation rates (Timm et al., 2022). Given the vulnerability of the study sample—characterized by low-socioeconomic status, caring for multiple children, a high proportion of single parents, high unemployment rates, and low education levels—caregivers might encounter additional barriers to engaging in the intervention. These barriers are likely driven by time and financial constraints (Ling et al., 2016) and contribute to the suboptimal average participation rate of 55.6%. Moreover, some caregivers expressed their hesitancy in participating in group discussions due to concerns about the appropriateness of their posts and low literacy. Addressing these challenges by offering flexible participation options (e.g., allowing make-ups), providing financial support (e.g., compensating for time and efforts), or adjusting participation expectations (e.g., encouraging positive responses to others rather than requiring original posts) may help mitigate these obstacles and enhance caregiver engagement.

Evaluations from both childcare teachers and caregivers affirm the acceptability of and satisfaction with the school-based mindfulness component. Young children gain knowledge through hands-on interactive experiences, so following the mindfulness principles to explore the connections between food and mind can foster a positive relationship with foods and enhance the formation of lifelong healthy eating habits (Gayoso et al., 2021). The calming effects of our mindful movement activities (i.e., yoga, breathing exercises) among preschoolers were endorsed by both childcare teachers and caregivers, supporting the promise of applying mindfulness to promote preschoolers’ emotional regulation and behavioral management (Pickerell et al., 2023; Sun et al., 2021). In addition, our child curricula were developed to be culturally and developmentally appropriate for preschoolers from LIEM families through actively engaging caregivers and childcare teachers during the development phase (Ling et al., 2016; Zahry & Ling, 2020). This study’s results further support this intention. Moreover, the approach of implementing a mindfulness-based lifestyle intervention by childcare teachers in real childcare settings has high potential for sustainability and scalability.

In addition to acknowledging that the school-based mindfulness component was fun and helpful, caregivers further endorsed their acceptance of and satisfaction with the home-based mindfulness component. Consistent with prior literature (Hammersley et al., 2020; Lawton et al., 2022), using the existing social media platform, Facebook, to engage caregivers into the intervention is feasible and acceptable to participants. The private Facebook group offered peer support and motivation. Although in-person engagement can result in better connections among participants, families from LIEM backgrounds (particularly in rural settings) face challenges, such as lack of time, childcare support, and reliable transportation, which prevent them from participating in in-person activities (Ling et al., 2016). However, due to individual differences in Facebook familiarity and use (Lawton et al., 2022), caregivers’ participation in interventions can vary. To accommodate most caregivers, a weekly intervention delivery frequency is recommended, considering that more than 90% of caregivers use Facebook at least weekly (Waring et al., 2023). Additionally, some parents may feel uncomfortable sharing personal information or engaging in discussions on a public platform due to increasing awareness of data privacy issues (Steijn et al., 2016), which could limit their engagement in the Facebook-based program. Offering a secure, private intervention website would provide a safe and more controlled environment, increasing trust and encouraging greater involvement in the intervention.

The virtual caregiver meeting also received positive feedback from caregivers. The acceptability of online yoga among caregivers was consistent with a prior study among adults with chronic conditions and their caregivers (Portz et al., 2023). The virtual format overcomes some barriers, such as lack of transportation, but it also presents new challenges, such as unreliable Internet services for caregivers to attend a group meeting. One previous healthy lifestyle intervention has achieved higher meeting attendance (51–87%) by providing each caregiver a tablet with reliable Internet services (Ling et al., 2018). Thus, providing this access needs to be considered when targeting LIEM caregivers, especially those in rural settings.

Caregivers considered the weekly motivational text messages to be motivating in their daily lives. Our intervention did not request a response from caregivers, in consideration of their participation burden and evidence that responses to text messages do not result in additional positive effects in improving behaviors or mental health (Figueroa et al., 2022). The texting time in this study was at noon, which was suggested as the best time for maximum engagement (Orred, 2024). Based on caregivers’ feedback, engaging them with weekly text messages may be a cost-effective way of ensuring retention during long-term follow-ups (Chaudhari et al., 2020).

Feedback from caregivers and teachers indicates the communication by letters from childcare to home is a positive experience for all parties. These findings align with prior studies (Ling et al., 2018, 2024a, 2024b) and support the promising effects of applying this method to encourage communication between school programs and home practices. The results also underscore the interdependence between preschoolers and caregivers, as demonstrated in the Actor–Partner Interdependence Model (Cook & Kenny, 2005). Consistent with the partner effects observed from children to parents in a prior study (Fowler et al., 2021), caregivers in this study shared that their food purchases and preparation behaviors were driven by their preschoolers’ attitudes and behaviors related to the intervention childcare experiences. Moreover, this child-led strategy helps establish a weekly routine, which can trigger more active communication from preschoolers to caregivers after school (Bradbard et al., 1992; Hosokawa et al., 2023). Enhancing school-home communication can improve home involvement in preschoolers’ learning (Lin et al., 2019). Therefore, letters can serve as an ongoing, low-cost feedback tool for promoting school-home communication, thereby improving interventions’ effectiveness and sustainability.

### Limitations

The study population is preschoolers and caregivers from LIEM families, potentially limiting its generalizability to middle- and high-income families. Moreover, since the intervention aimed to modify eating behaviors and body movement, we excluded preschoolers with motor disabilities or impairments, medical conditions restricting dietary changes, or developmental disorders affecting communication. However, this exclusion may have inadvertently omitted those with the greatest need. The intervention introduced preschoolers in childcare centers to a variety of fruits and vegetables, but encouraging caregivers to provide fresh produce at home could impose a financial burden on participating families. Sharing information on community resources and food assistance programs may help alleviate this challenge.

The U.S. Household Food Security Survey Module had low reliability of 0.57 in this study. A prior study with 149 adults also found a low Cronbach’s alpha of 0.62 (Md Isa et al., 2016). The low reliability may be due to item redundancy between the adult and child survey modules, inconsistent response choices between items, or a small sample size. While the sample size was adequate for exploring the feasibility of the study and intervention, further validation in a larger-scale study is necessary. The study’s evaluation period was limited to 5 weeks, which may have minimized the potential time effects that may occur in the full 16-week intervention, leading to decreased engagement from caregivers over an extended period. Additionally, due to the developmental stage of young children, data were not collected to evaluate their acceptability and satisfaction with the school-based component. It is certainly possible that preschoolers’ impressions may differ from those of caregivers and teachers, and there could be potential biases in the reports provided by caregivers and teachers. In addition, long-term follow-up and cost evaluation are suggested to assess the intervention’s sustainability and cost-effectiveness.

## Conclusions

Results from this study indicate strong feasibility, acceptability of, and satisfaction with the 5-week mindfulness-based lifestyle intervention among preschoolers, caregivers, and childcare teachers within both urban and rural contexts. The implementation of various strategies, such as accommodating participants’ communication preferences and specific needs, implementing a structured reminder system, engaging community stakeholders, offering monetary incentives, and providing effective teacher training, successfully enhanced participant recruitment and participation. Focusing on mindfulness to improve preschoolers’ MEB health may be a promising approach to help achieve optimal child development by leveraging resources both at school and at home.

## Supplementary Information

Below is the link to the electronic supplementary material.

Supplementary file1 (DOCX 19 KB)
