# Supplementary material for: A Mindfulness-Based Lifestyle Intervention Among Economically Marginalized Caregiver-Preschooler Dyads: Feasibility, Acceptability, and Satisfaction
Source: School Ment Health. 2025 May 26;17(2):715–32. doi: 10.1007/s12310-025-09767-w (PMC12241280; doi:10.1007/s12310-025-09767-w)
Supplement: Supplementary file 1 — Supplementary file1 (DOCX 19 KB) [file 12310_2025_9767_MOESM1_ESM.docx]

**Caregiver Semi-Structured Interview Guide**

Thanks for taking the time to share your opinions with us. Keep in mind that we're just as interested in negative comments as positive comments, and at times the negative comments are the most helpful. We're tape recording the session because we don't want to miss any of your comments. Information collected from this session will be used by the researchers at Michigan State University to help them plan future programs. Information will only be presented in presentations or publications in group form. No individual names or identifiable information will ever be listed with a participant’s response.

**OPENING QUESTION:**

To start with, please tell me your overall experience participating in the program with your Head Start child.

PROBE: What are the activities you like most?

PROBE: What are the barriers/challenges that prevented you from participating in some activities?

**RECRUITMENT:**

What do you think about the recruitment flyer?

Are the contents appropriate?

What suggestions do you have to make the flyer more attractive?

Who do you think should approach you about the study?

When the flyers should be sent out to you?

How should the recruitment flyers be sent out to parents?

What recruitment strategies do you think will work best to recruit parents?

**PARENT MEETINGS:**

Now let us talk about the virtual parent meetings, please tell me what you like about the parent meetings.

What do you dislike about the parent meetings?

What about the meeting discussion contents? (e.g., mindful eating at Meeting 1; tai chi and mindful walking at Meeting 2; mindful communication and deep breathing exercises at Meeting 3)

What about the 20-minute yoga session at each meeting? What are your suggestions to improve it?

What do you think of the program cookbook? What other recipes do you want?

What are the barriers that prevented you from participating in the parent meetings?

What support do you need in order to attend the parent meetings in the future?

How can we make the parent meetings better together (format: face-to-face or virtual, location, discussion topic, duration, days of the week, time of the day)?

**SOCIAL MEDIA-BASED PROGRAM:**

Please tell me what you like about the online parent program.

What do you dislike about the online parent program?

What are your comments on the weekly flyers and videos posted?

What do you think about the weekly tasks and quiz (content, number, frequency)?

What are the barriers that prevented you from completing the weekly tasks or quiz?

How do others’ posting (type of post/content and frequency) influence your behaviors and health?

What else should we include in the online parent program to help you create a mindful and healthy family environment for your child?

How can we together make the online parent program better?

**MOTIVATIONAL TEXT MESSAGING:**

How do you like the motivational text messages you received each week?

What are your thoughts on the frequency, content, and sending time?

How helpful are these text messages to you?

What do you think we send you one weekly motivational text message on Monday noon in the following 12 months?

Do you want to continue receiving motivational text messages?

**CHILD LETTER TO PARENTS:**

What do you think about your child’s letters?

How much has your child shared with you about the school program?

How does child’s participation (child letters) influence what you are doing at home related to mindful eating and movement?

What can we do to make the child letters more effective?

Any comments on the school program for your child?

**ENDING QUESTION:**

How does the program in general influence your family’s thoughts and behaviors about promoting physical and mental health?

Before we end our discussion, are there other thoughts, comments, or suggestions that you would like to share with me to improve our children’s overall health, or to help us successfully involve parents in this type of research study?

**ENDING COMMENTS:**

Thank you very much for agreeing to participate in this discussion today. We really appreciate your time to share with us your thoughts and ideas.

**Teacher Semi-Structured Interview Guide**

Thanks for taking the time to share your opinions with us. Keep in mind that we're just as interested in negative comments as positive comments, and at times the negative comments are the most helpful. We're tape recording the session because we don't want to miss any of your comments. Information collected from this session will be used by the researchers at Michigan State University to help them plan future programs. Information will only be presented in presentations or publications in group form. No individual names or identifiable information will ever be listed with a participant’s response.

**OPENING QUESTION:**

To start with, please tell me your classroom’s overall experience participating in the program.

PROBE: What are the activities you like most?

PROBE: What are the barriers/challenges that prevented your classroom from participating?

How does the program in general influence your classroom children’s physical and mental health?

What changes have your observed in your classroom’s children due to participating in the program?

**RECRUITMENT:**

What do you think about the recruitment flyer?

Are the contents appropriate?

What suggestions do you have to make the flyer more attractive?

Who do you think should approach parents about the study?

When the flyers should be sent out to parents?

How should the recruitment flyers be sent out to parents?

What questions did the parents ask about the study or recruitment flyer?

How can we facilitate the recruitment?

What recruitment strategies do you think will work best to recruit parents?

**CHILD PROGRAM:**

What do you think about the teacher training on delivering the program?

How can we improve the teacher training? (format: face-to-face or virtual, location, training content, duration, time of the year)?

What do you think about the child “Eat My ABCs” curriculum? (content age appropriate, curriculum book easy to follow, lesson duration reasonable)

What do you think about the child “Walk My ABCs” curriculum? (content age appropriate, curriculum book easy to follow, lesson duration reasonable)

What are the challenges you have when teaching the lessons?

What are your suggestions to effectively teach mindful eating and movement to young children?

What do you think about helping children to complete the weekly child’s letters using stickers for parents?

How can we make the child letters more effective?

How can we improve the overall child program?

What support do you need to incorporate the program into your classroom daily routine?

**PARENT PROGRAM:**

Now let us talk about the parent component.

Overall, what are your thoughts of our parent program including the social media-based component and parent group meetings?

Based on your experience, how can we effectively engage parents in promoting their children’s health? Via group meetings, online program?

How can we improve the overall parent program?

**ENDING QUESTION:**

What other thoughts, comments, or suggestions you would like to share to improve our children’s overall health, or to help us improve our program?

What health promotion programs do you think your classroom’s children need to foster a healthy future?

What health promotion programs are you interested in participating along with your classroom’s children?

What support/assistance do you need to participate in a program such as the Food-Body-Mind program?

**ENDING COMMENTS:**

Thank you very much for agreeing to participate in this discussion today. We really appreciate your time to share with us your thoughts and ideas.
